# *Staphylococcus aureus* Infection and Persistence in Chronic Rhinosinusitis: Focus on Leukocidin ED

**DOI:** 10.3390/toxins12110678

**Published:** 2020-10-28

**Authors:** Dimitri Poddighe, Luca Vangelista

**Affiliations:** 1 Department of Medicine, Nazarbayev University School of Medicine, Nur-Sultan 010000, Kazakhstan; dimitri.poddighe@nu.edu.kz; 2 Department of Pediatrics, National Research Center for Maternal and Child Health, Nur-Sultan 010000, Kazakhstan; 3 Department of Biomedical Sciences, Nazarbayev University School of Medicine, Nur-Sultan 010000, Kazakhstan

**Keywords:** *Staphylococcus aureus*, chronic rhinosinusitis (CRS), persistent infection, nasal polyps, LukED, CCR5

## Abstract

Chronic rhinosinusitis (CRS) is thought to be a multifactorial disease that includes a direct involvement of bacteria that trigger inflammation and contribute to CRS pathogenesis. *Staphylococcus aureus* infection and persistence is associated with chronic rhinosinusitis (CRS), and it may be particularly relevant in the form with nasal polyps (CRSwNP). The large array of exotoxins deployed by *S. aureus* is instrumental for the bacterium to warrant its infection and dissemination in different human body districts. Here, we analyze the common Th2 environment in CRSwNP and prospect a possible dynamic role played by *S. aureus* leukocidins in promoting this chronic inflammation, considering leukocidin ED (LukED) as a strong prototype candidate worth of therapeutic investigation. CCR5 is an essential target for LukED to exert its cytotoxicity towards T cells, macrophages and dendritic cells. Therefore, CCR5 blockade might be an interesting therapeutic option for CRS and, more specifically, persistent and relapsing CRSwNP. In this perspective, the arsenal of CCR5 antagonists being developed to inhibit HIV-1 entry (CCR5 being the major HIV-1 co-receptor) could be easily repurposed for CRS therapeutic investigation. Finally, direct targeting of LukED by neutralizing antibodies could represent an important additional solution to *S. aureus* infection.

## 1. Introduction

The term “sinusitis” refers to inflammation in the nasal sinuses. Since it is usually associated with the inflammation of nasal mucosa (namely, rhinitis), the term “rhinosinusitis” is currently adopted. The clinical manifestations of rhinitis and rhinosinusitis are often similar, and, thus, the main discriminatory criterion is the symptom duration, which is usually longer than 7–10 days in rhinosinusitis. Acute rhinosinusitis is defined by the resolution of sinus-nasal symptoms within four weeks; if these symptoms persist longer, but for no more than 12 weeks from the onset, this clinical condition is reported as subacute rhinosinusitis; finally, if the sinus inflammation and the related clinical manifestations persist longer than 12 weeks, this situation is named chronic rhinosinusitis (CRS) [1,2,3].

CRS is an important health problem in terms of long-term morbidity and quality of life, in addition to being a financial burden for the patient and/or the health system. CRS is diagnosed in both adults and children. Although pediatric and adult CRS are different in many aspects (e.g., incidence, anatomic pathology, predisposing factors and therapeutic aspects), several immunopathologic aspects are similar and can be summarized in two main categories: CRS with nasal polyps (CRSwNP) and without nasal polyps (CRSsNP) [3,4,5]. CRSsNP is characterized by an inflammatory infiltrate enriched in neutrophils deriving from a Th1 immune response, where IL-1, IL-6, IFNγ and TNFα are the main pro-inflammatory cytokines. It usually results from an acute/subacute “bacterial” sinusitis that could not be resolved completely. CRSwNP shows a persistent inflammation dominated by eosinophilic infiltrates, which are orchestrated by Th2 cytokines (e.g., IL-4, IL-5 and IL-13). Tissue eosinophilia, sometimes associated with eosinophilia in peripheral blood, correlates to CRSwNP severity [6,7,8].

CRS is thought to be a multifactorial disease process in which bacteria are supposed to trigger, promote or maintain the inflammation. Indeed, a polymicrobial flora is constantly described in patients affected with CRS. In general, *Staphylococcus aureus*, *S. epidermidis* and anaerobic Gram-negative bacteria predominate in CRS, but the exact composition varies according to the clinical context (presence of comorbidity), age and anatomical location [8,9]. The studies reported so far show a polymicrobial aerobic-anaerobic flora that is not qualitatively different between CRSwNP and CRSsNP. However, the colonization rate of *S. aureus* increases in patients with CRSwNP and correlates to the percentage of eosinophils in the mucosal infiltrate and in the peripheral blood [10]. Moreover, a meta-analysis showed a greater rate of *S. aureus* culture-positivity in the CRSwNP group compared to the control group [11]. Several independent studies showed higher rates of upper airways *S. aureus* colonization in CRSwNP patients, which correlated with disease severity [12,13,14]. One of these studies reported nasal cavity *S. aureus* colonization in 64% of patients with nasal polyps, whereas *S. aureus* was present in only 33% and 20% of CRSsNP subjects and healthy controls, respectively [13].

Therefore, *S. aureus* is thought to play a specific role in the immunopathogenesis of CRS, and its contribution may be even larger in CRSwNP, especially in relapsing cases. In addition to biofilms, *S. aureus* is known to secrete several toxins, which may contribute to its persistence and role in the setting of CRSwNP. Here, we focus on the specific action of leukocidins and propose leukocidin ED (LukED) as a prototype target in CRS, given the ability of LukED to kill several immune cell types and the availability of numerous compounds that can block its interaction with CCR5.

## 2. *Staphylococcus aureus* Toxins

Depending on the host-pathogen interaction, *S. aureus* may engage the host with an arsenal of different exotoxins aimed to achieve different goals, including the neutralization of the immune system response against the bacterium, thus favoring invasion and persistence. Although different isolates of *S. aureus* produce only a portion of all existing *S. aureus* toxins, they have the ability to significantly challenge the host. Within the formidable array of *S. aureus* exotoxins, many members remain ill-characterized for their role in staphylococcal pathology [15].

Human-specific *S. aureus* exotoxins fall into three main categories: pore-forming toxins, enzymatic toxins and superantigens. The pore-forming toxins include α-toxin and bi-component leukocidins (LukSF-PV, HlgAB, HlgCB, LukED and LukAB) that act by initial recognition of a receptor determinant on the surface of the target cell, followed by oligomerization (α-toxin forms heptamers and leukocidins form octamers) and pore formation. Other toxins include ε-toxin, which causes the lysis of keratinocytes, and phenol-soluble modulins, which are responsible for cell lysis, biofilm formation and immune modulation. Furthermore, *S. aureus* secretes a large number of enzymes; these include β-toxin (sphingomyelinase), exfoliative toxins (serine proteases) and several cofactors and exoenzymes that serve the purpose of bacterial survival and dissemination. Many *S. aureus* exoenzymes participate in biofilm formation, disruption and remodeling. Staphylococcal superantigens are divided in a large number of T superantigens (SAgs) and one B cell superantigen, staphylococcal protein A (SpA). Superantigens exert their toxicity by promoting a massive activation of T or B cell repertoires that leads to clonal deletion, ultimately compromising adaptive immunity [15].

Antibiotic resistance is increasing worldwide and includes methicillin-resistant *S. aureus* (MRSA). MRSA strains express several exotoxins, and, among them, LukED has been found in 88–99% of the isolates of different geographical locations [16]. The prevalence of MRSA strains is also increasing among CRS patients [17], making the option for treatments alternative to antibiotics more urgent over time.

## 3. *Staphylococcus aureus* Toxins in Chronic Rhinosinusitis with Nasal Polyps

Eosinophil-dominant inflammatory infiltration is found in most CRSwNP patients (70–90%), and is the expression of a Th2-polarized immune response [6,8]. Unlike CRSsNP, CRSwNP is often complicated by allergic disorders such as asthma, aspirin intolerance and other drug allergies, in addition to a frequent association with allergic rhinitis [2,5]. Therefore, the Th2-mediated eosinophilic inflammation in CRSwNP is thought to be driven by a persistent allergenic stimulation that leads to elevated production of IL-4, IL-5, IL-13 and IgE. Even though the allergens triggering this pathological condition have not been elucidated, bacteria determinants, such as in *S. aureus*, are considered potential candidates [8,18]. *S. aureus* enterotoxin superantigens are supposed to play a role in the allergenic sensitization underlying CRSwNP [8]. Bachert et al. showed that, in approximately 50% of CRSwNP patients, the homogenates derived from nasal polyps contained IgE antibodies specific to *S. aureus*-derived enterotoxins (SAEs) A and/or B [19]. These patients were characterized by a strong eosinophilic infiltration, where Th2 cytokines resulted to be upregulated, in addition to an increased concentration of plasmatic IgE [19,20]. The same authors reported that *S. aureus* colonization was present in 60–65% of CRSwNP patients and in 87.5% of the subgroups with comorbid asthma and/or aspirin sensitivity. These colonization rates were significantly different from those found in controls and in the CRS population (33.3% and 27.3%, respectively). The prevalence of IgE antibodies compared to SAEs in tissue homogenates was found to parallel the colonization rates reported above. Importantly, SAE-specific IgE antibodies were rarely found in CRSsNP, further supporting the immunopathogenic difference from CRSwNP [12]. In addition to the production of SAE-specific IgE, many CRSwNP patients colonized by *S. aureus* presented polyclonal IgE antibodies both locally and systemically [21,22]. It has been suggested that SAEs can influence local T cell diversity. T lymphocytes localized in nasal polyp tissues seem to have a higher T cell receptor V-beta expansion compared to circulating T lymphocytes, which can enhance polyclonal IgE production and substantially contribute to persistent Th2 inflammation [23,24]. Recently, Altunbulakli et al. found that the epithelial barriers of nasal polyps in CRSwNP patients are disrupted because of a defective expression of tight junctions [25]. However, epithelial barrier leakiness has been shown to correlate with an increased level of pro-inflammatory cytokines in the nasal polyps, rather than being a direct consequence of exoenzymes produced by *S. aureus* [25]. Serine protease-like protein (Spl) D, one of the six subtypes (SplA-SplF) produced by *S. aureus*, can increase the production of IL-33, promoting the development of Th2 immune responses [26,27].

An important clinical aspect of CRSwNP is the high rate of relapse, even after endoscopic sinus surgery, required in at least 30% of patients to restore patency and aeration to the sinuses [13]. According to a very recent systematic review by Chen et al., the percentage of CRSwNP patients undergoing surgery may considerably vary depending on the geographic region (43–84%), and 21–59% of these patients were reported to need revision surgery [28].

Therefore, the factors promoting the persistence and immune evasion of *S. aureus* in CRSwNP patients may be as important as those creating the Th2-biased immune environment. In addition to the production of biofilms that prevent antibiotic penetration and protect *S. aureus* from recognition by the host immune system, exotoxins acting on immune cells (such as leukocidins) may play a significant pathogenic role.

## 4. LukED as a Possible Pathogenic Factor in CRSwNP and Prototype for Therapeutic Intervention

*S. aureus* can secrete cytolytic toxins to injure immune cells, namely leukocidins [29]. Among them, LukED resulted to be the leukocidin with the widest tropism among human white blood cells.

As they provide resistance and thus persistence to *S. aureus*, microbial biofilms have been implicated in the pathogenesis of CRSwNP [30,31]. A recent study by Cirkovic et al. specifically assessed the biofilm production in patients with CRSwNP, and *S. aureus* resulted to be a stronger biofilm producer compared to other bacterial species present in patients’ polymicrobial flora [32]. However, the same authors provided robust in vitro evidence that topical steroids, nasal saline and antibiotics (amoxicillin-clavulanic acid and levofloxacin), the mainstays of CRS medical management, are potent anti-biofilm agents in patients with CRSwNP [32,33]. Therefore, we might speculate that additional factors (along with the development of antibiotic resistance and the production of biofilms) could contribute to the persistence of *S. aureus* in CRSwNP and its recurrence after surgical procedures. Immune evasion is supposed to be one of those factors, and LukED may cause a profound and local impairment of local host immunity against *S. aureus*, due to its capacity to injure several immune cells participating in both innate and adaptive immune responses. We therefore wish to focus on LukED as a prototypic target for combating *S. aureus* infection and establishment in CRSwNP patients.

LukED is a relatively recently discovered leukocidin [34]. Common to bi-component leukocidins, LukE and LukD share a similar fold composed of a β-sandwich divided into three domains: the cap, the rim and the stem [35]. The conformation of these domains remains largely unmodified within the pre-pore octamer on the surface of the target cell, and specific inter-component cap-cap and rim-rim interfaces mediate oligomerization [35]. In its mechanism of action, LukED first recognizes chemokine receptors (CCR5, CXCR1, CXCR2 and DARC) on the surface of target cells (monocytes, macrophages, dendritic cells, T cells, NK cells and erythrocytes) [29]. This initial docking is mediated by the LukE component that presents different molecular determinants within its rim domain loops that are specific for the recognition of the different chemokine receptors. Five sequence divergent regions (DR) between LukE and LukS-PV rim domains have been identified; within LukE, DR1 is the moiety determining CCR5 recognition [36], while DR4 is responsible for CXCR1 and CXCR2 binding [37]. In addition, differential post-translational modifications of leukocidin receptors determine individual variability in susceptibility to *S. aureus* infection [38]. Following chemokine receptor engagement, the LukD component joins cell-bound LukE, and oligomerization of the toxin initiates on the surface of the target cell, ending with the formation of a bi-component LukED octamer. Finally, a remarkable conformational change within the stem domain of the LukE and LukD components within the octamer leads to the formation of a β-barrel pore that spans the membrane, unleashing LukED cytotoxic activity [29]. The large array of different immune cell types targeted by LukED makes it a particularly deadly toxin. In a mouse model of infection, LukED has been reported to be a lethal *S. aureus* virulence factor for wild type, as compared to CCR5 knockout, animals [39]. CCR5 is therefore a very important target for the development of therapeutics to counteract *S. aureus* infection. Coincidentally, CCR5 is best known for being the major co-receptor that HIV-1 uses for its entry into target cells, namely, the same cells targeted by LukED in its CCR5 tropism, i.e., T cells, dendritic cells and macrophages. In this line of thought, the inhibition of the interaction between LukE and CCR5 can be tackled using the armamentarium developed for the inhibition of HIV-1 entry [40]. The most immediate candidate to block LukED targeting of CCR5^+^ cells is maraviroc, a small chemical CCR5 antagonist that is FDA-approved as an HIV-1 entry inhibitor that has already been reported to efficiently inhibit LukED [39]. Other CCR5 antagonists amenable to tackling LukED are the latest derivatives of CCL5 (a natural chemokine ligand of CCR5) that proved to be extremely potent HIV-1 inhibitors in cellular assays [41]. There are also three monoclonal antibodies that act as CCR5 antagonists and HIV-1 blockers that could therefore possibly block LukED: PRO 140, CCR5mAb004 and RoAb13 [40].

Unfortunately, there is no study that investigated the specific role played by leukocidins in patients affected with CRSwNP (and more generally CRS). However, Bardy et al. investigated and compared the distribution of membrane damaging *S. aureus* toxins in CRSwNP and CRSsNP patients; interestingly, both LukD and LukE resulted to be more prevalent in the former cohort (67% vs. 57%, and 77% vs. 54%, respectively). However, only a single colony of *S. aureus* was selected for sequencing from each patient, and a larger dataset would have strengthened the statistical significance of their results [42].

Currently, there is no evidence suggesting a role for LukED in shifting the adaptive immune response toward a Th2 profile. However, Alonzo et al. showed that the treatment of blood lymphocytes with LukED resulted in the depletion of most effector memory T lymphocytes [39]. Importantly, the CCR6^+^CCR5^+^ memory T cell subset was previously demonstrated to produce more IL-17 and IFN-γ than CCR6^+^CCR5^−^ cells [43]. Indeed, Alonzo et al. demonstrated that the depletion of CCR5^+^CD4^+^ T cells by LukED significantly reduced the proportion of IFN-γ and IL-17-producing cells, thus impairing the Th1 and Th17 immune responses [39]. This may concomitantly impair the bacterial clearance and skew the immunological environment in the sinus mucosa towards a Th2 phenotype.

Absent or reduced expression of CCR5 confers resistance to LukED and possibly to *S. aureus* infections [44]. Individuals carrying a 32 base-pair deletion in the CCR5 gene (CCR5Δ32) produce a non-functional receptor that cannot reach the cell surface, and their T cells are resistant to LukED [39]. Interestingly, de Souza et al. concluded that CCR5Δ32 carriers could be more protected against osteomyelitis caused by *S. aureus* [45]. Even though there are no studies exploring LukED-CCR5 interplay in CRS, the role of CCR5 in bacterial and parasitic infections should be considered [40,46].

A very recent report presented the discovery of C_14_PC, a small chemical compound capable of neutralizing several leukocidins (namely, LukED, LukSF-PV and α-toxin) that dramatically contribute to MRSA pathogenesis. C_14_PC exerts its action by intercalating in the conserved phosphocholine binding groove in the α-toxin protomer, LukF-PV and LukD, thus proving to be a promising inhibitor of leukocidins and a valid alternative to antibiotics [47].

An important alternative to blocking LukED is represented by its direct targeting in a vaccine scenario. A very recent report demonstrated that antibodies raised against leukocidins (including LukED) can protect against *S. aureus* bacteremia [48].

## 5. Conclusions

In conclusion, if a specific and significant contribution of LukED to the resistance and persistence of *S. aureus* in patients with CRSwNP and its relapsing forms might be demonstrated, the availability of a CCR5 antagonist such as maraviroc, chemokine derivatives and monoclonal antibodies might open the road to speculate on a potential use for these molecules in clinical settings, although effectiveness and safety should be assessed in each specific clinical scenario. Finally, direct targeting of leukocidins, and thus LukED, by neutralizing antibodies could represent the solution to the long sought vaccine against *S. aureus*.
